# Solid‐Like yet Reconfigurable 3D‐Printed Liquid Tubular Wires From Nonconductive Molecules

**DOI:** 10.1002/advs.202524287

**Published:** 2026-03-09

**Authors:** Yuchen Fu, Weixi Wu, Wei Chen, Jiangang Zhang, Biyu Jin, Sai Zhao, Yu Chai

**Affiliations:** ^1^ Department of Physics City University of Hong Kong Kowloon Hong Kong SAR China; ^2^ City University of Hong Kong Shenzhen Research Institute Shenzhen China; ^3^ Materials Science and Engineering Program and Texas Materials Institute The University of Texas at Austin Austin Texas USA

**Keywords:** 3D printing, conductive, interfacial reaction, liquid interface, reconfigurable electronics

## Abstract

Electronic devices composed entirely of liquids offer numerous advantages, including self‐healing of mechanical fractures, the ability to conduct electrical currents, and the capacity to transduce mass via liquid flow, making them a promising alternative to conventional solid‐state electronics. A key step toward fully liquid electronics is the fabrication of all‐liquid wires that support efficient current flow while maintaining a well‐defined, solid‐like shape. However, early demonstrations have been limited by relatively low electrical and mechanical performance and a narrow range of intrinsically conducting materials, hindering broader applications. Here, we demonstrate an interfacial redox strategy to produce all‐liquid, 3D conducting wires. The interfacial assembly relies on the polymerization of nonconductive monomers at the liquid‐ink‐bath interface. Benefiting from in situ polymer network formation, the liquid–liquid interface attains high interfacial stiffness while enabling continuous 3D printing, yielding the rather stiff 3D‐printed all‐liquid tubular wire. To further establish feasibility, we systematically investigate interfacial assembly and mechanical properties, evaluate 3D printing performance, and demonstrate functional electronic devices that incorporate our 3D‐printed all‐liquid tubular wire. This study introduces a novel method for fabricating conductive all‐liquid electronics from nonconductive materials, demonstrating its potential for advancing next‐generation electronic devices.

## Graphical abstract

This work shatters the current reliance of liquid electronics on intrinsically conductive materials by introducing a novel interfacial redox strategy that transforms non‐conductive molecules into high‐performance, 3D liquid tubular wires with solid‐like electrical properties and exceptional reconfigurability. It opens a vast design space for all‐liquid electronic components.

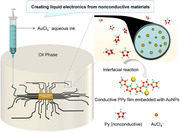

## 1 Introduction

Reconfigurable electronics are increasingly in demand for soft robotics, wearable systems, and biomedical interfaces, where devices must adapt their form and function to dynamic, complex environments [1, 2, 3, 4]. Conventional solid‐state platforms—even soft variants—are reliable but inherently limited in reconfigurability, self‐repair, and geometric compliance [5]. By contrast, liquid‐based electronics deform without fracture, self‐heal after damage, and conform to intricate 3D geometries, making them attractive for next‐generation adaptive devices [6]. Yet, despite this promise, fully all‐liquid electronic systems remain elusive. A fundamental building block of such systems is the all‐liquid wire: a structure that conducts current while retaining a defined, solid‐like shape, yet preserves the reconfigurability and mass‐transducing capabilities of liquids. Prior approaches, leveraging interfacial assembly and jamming of conductive species such as liquid metals, conductive polymers, and ionic liquids [7, 8, 9, 10, 11, 12], have demonstrated feasibility but suffer from limited material choice, narrow printing windows, and insufficient mechanical stiffness, constraining their scalability and robustness [5, 13, 14].

Here, we overcome these constraints by decoupling mechanical shape retention from the intrinsic conductivity of interfacially active species. Using interfacial redox chemistry, we 3D print liquid wires from nonconductive monomers that polymerize in situ at the ink‐bath interface to form a robust interfacial network. This interfacial polymer layer stabilizes the printed geometry while, owing to its large effective molecular size, avoiding the substantial reduction in interfacial tension typically caused by small‐molecule surfactants. Simultaneously, the in situ formation of the interfacial network enables tunable electrical properties. Consequently, our all‐liquid wires exhibit high printing fidelity, strong mechanical stiffness, and excellent electrical conductance.

Notably, unlike conventional metallic conductive wires (e.g., liquid metals, polymer‐metal composites) that conduct electricity throughout their entire volume [15, 16], our all‐liquid wires exhibit a distinct core‐shell architecture: electrical transport occurs primarily through the conductive outer shell formed at the interface, while the inner liquid core remains nearly nonconductive. To distinguish this architecture, we term our printed structures liquid tubular wires. Although metallic conductors may offer higher intrinsic conductivity, the interface‐formed conductive film of the liquid tubular wire uniquely combines electrical conductivity with mechanical stability, acting simultaneously as a current‐transport medium and a structural scaffold that enables self‐supporting 3D liquid wiring. This strategy inherently avoids the brittle fracture typical of solid filler‐based composites and eliminates leakage risks associated with liquid metals [17, 18]. Moreover, it provides dynamic reconfigurability that enables on‐demand severing and reconnection, capabilities unattainable in metallic conductive wires. As a model system, we use pyrrole, which undergoes interfacial oxidation by tetrachloroaurate (AuCl_4_^−^) to yield a polypyrrole (PPy) coating with concomitant gold nanoparticle formation at the ink–bath interface. This interfacial assembly provides solid‐like structural integrity with liquid‐phase reconfigurability, establishing a general and promising route toward practical, fully liquid electronic components.

## 2 Results and Discussion

### 2.1 In Situ Formation of the Conductive Interfacial Film

**FIGURE 1:**
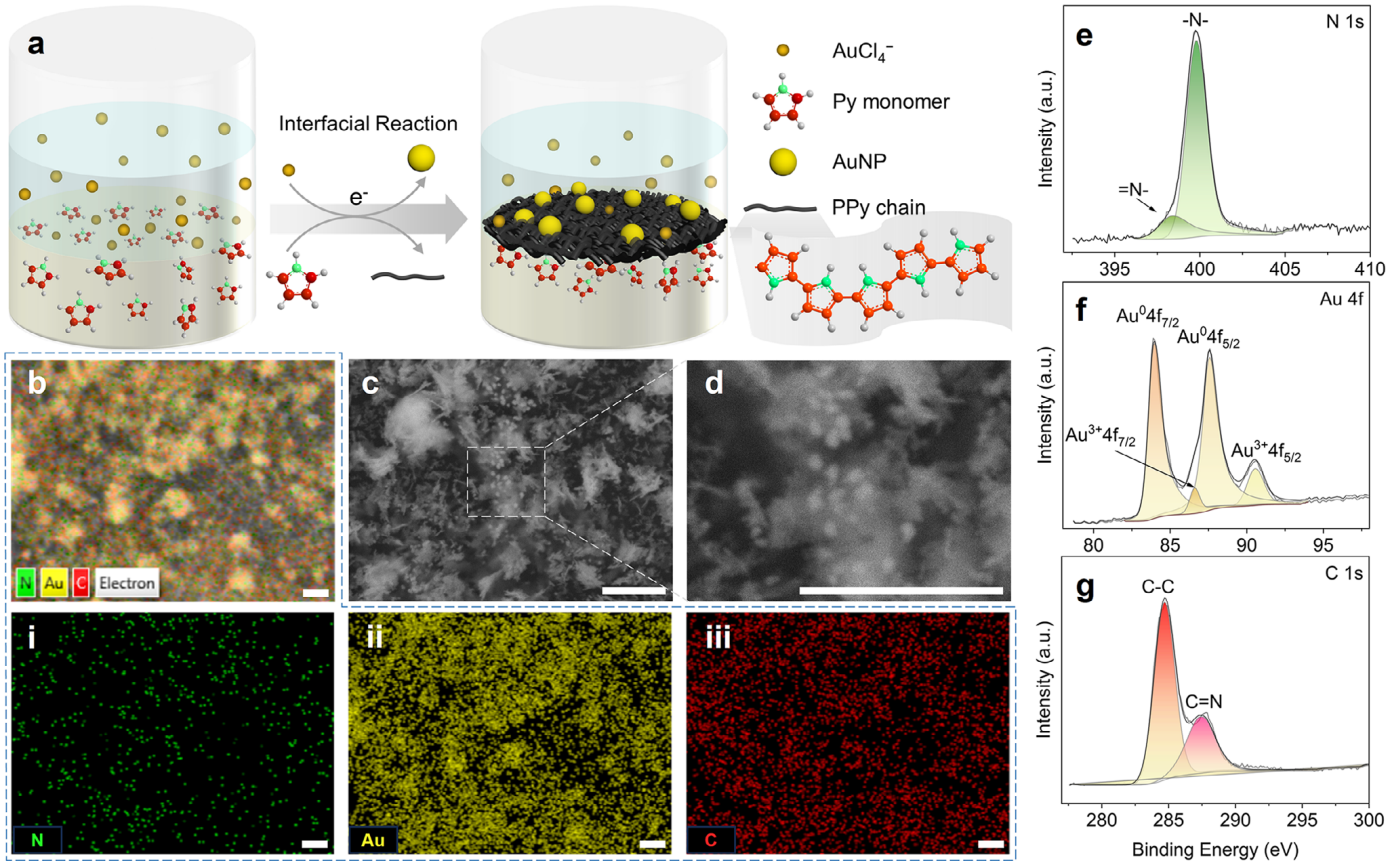
In situ formation of the interfacial conductive film and its surface characterizations. (a) Schematic of the formation process of the PPy film at the water–oil interface by interfacial redox reactions. (b) Element mapping images of N (i), Au (ii), and C (iii) of the formed interfacial film by EDS analysis. SEM images of (c) the resultant interfacial film, and (d) its magnified region. The high‐resolution XPS spectra of (e) N 1s, (f) Au 4f, and (g) C 1s. Scale bar, 1 µm.

A PPy film embedded with gold nanoparticles (AuNPs) forms in situ at the liquid ink and bath interface (water–oil) via interfacial redox polymerization. As illustrated in Figure 1a, aqueous tetrachloroaurate (AuCl_4_^−^) oxidizes pyrrole (Py) monomers to radical cations, which subsequently couple and propagate into conductive PPy chains; subsequently, AuCl_4_^−^ is reduced to metallic gold. This interfacial reaction yields a cohesive polymer network that imparts solid‐like integrity to the liquid–liquid interface while introducing a conducting pathway.

Scanning electron microscopy (SEM) confirms the evolution of the interfacial architecture (Figure 1b–d). Early‐stage, rim‐like fibrils nucleate at the interface, grow, and interconnect into larger, ramified clusters consistent with a PPy network. Discrete bright spheres, randomly distributed within the polymer matrix, exhibit an average diameter of ∼80 nm and are assigned to AuNPs (Figure 1d). Energy‐dispersive X‐ray spectroscopy (EDS) maps further corroborate the composition: continuous nitrogen and carbon signals delineate the PPy network, while widespread gold signals indicate metallic nanoparticles together with residually adsorbed Au species (Figure 1b (i–iii)).

X‐ray photoelectron spectroscopy (XPS) provides further evidence of the interfacial redox process. The survey scan spectrum confirms the presence of N, C, Au, and O on the film surface, where N (399.89 eV) and C (285.18 eV) are characteristic of PPy, and Au (87.82 eV) corresponds to gold particles or ions (Figure S1). The O peak at 531.82 eV is most likely due to over‐oxidation from dissociative oxygen. High‐resolution spectra of each element offer deeper insights into the reaction process. The coexistence of ─N─ (at 399.43 eV) and ═N─ (at 398.02 eV) is found in the N 1s spectrum (Figure 1e), the former of which is dominated and assigned to pyrrolic nitrogen [19]. In the high‐resolution spectrum of Au 4f, the peaks at 83.95 and 87.57 eV are assigned to Au^0^4f_7/2_ and Au^0^4f_5/2_ [20, 21], indicating the complete reduction of the gold ions (Figure 1f; Table S1). It is also noted that two negligible peaks at 86.60 and 90.55 eV correspond to Au^3+^ [21], showing the insufficient reduction of these ions owing to the excess addition of AuCl_4_^−^ in the reaction system (Figure 1f; Table S1). This also explains the distribution of Au in Figure 1b (ii). Furthermore, the decomposition of the C 1s spectrum presents the C─C bond (at 284.8 eV) and the C═N bond (at 287.5 eV) as featured in the PPy patterns (Figure 1g) [19].

### 2.2 Dynamic Interfacial Formation of Mechanically Stiff PPy Film via Redox Reactions

**FIGURE 2:**
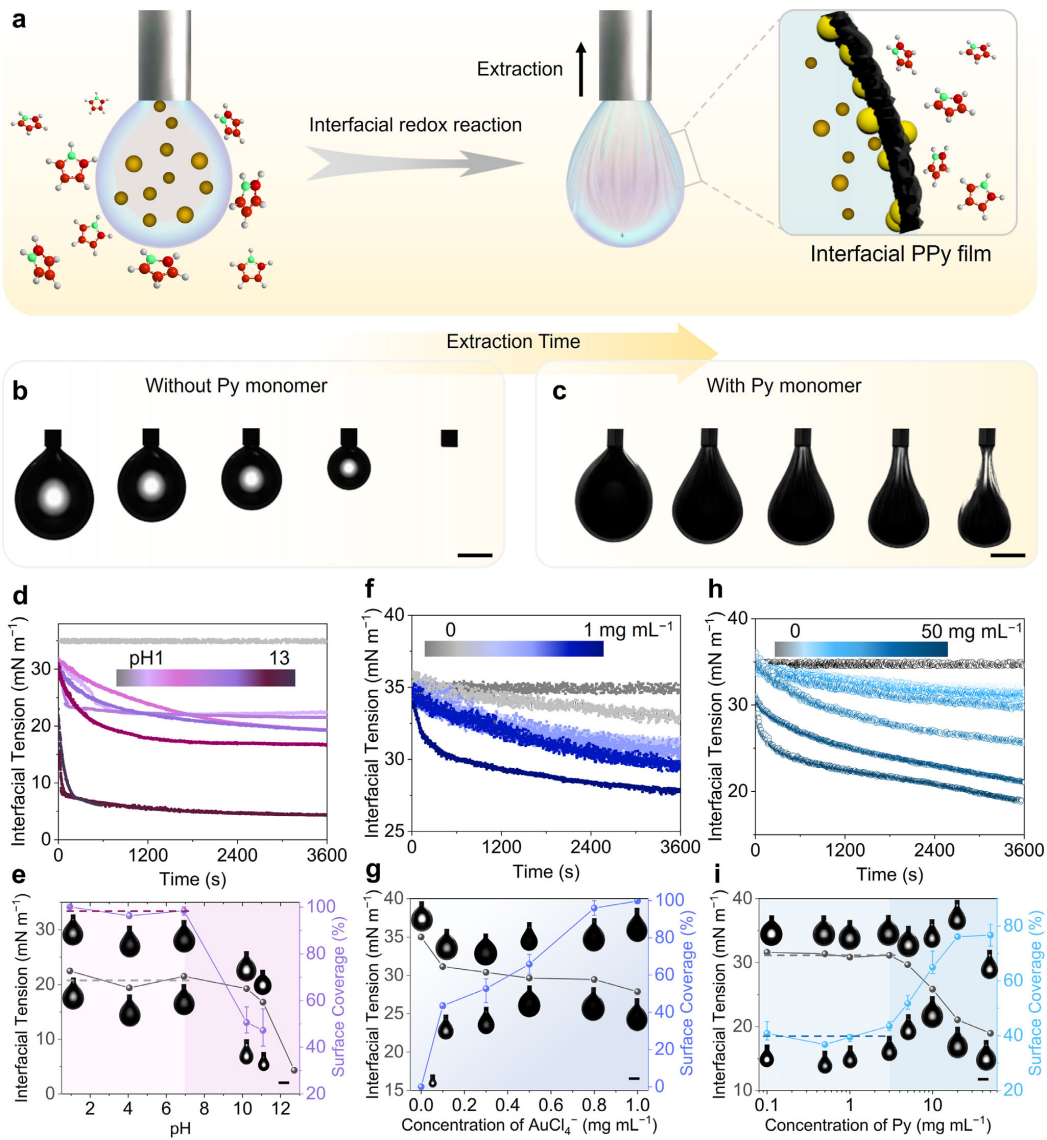
Interfacial behaviors of the film‐formed interface under different conditions. (a) Schematic of film formation at the droplet surface by interfacial redox reactions. Snapshots of the pendant droplet of 0.8 mg mL^−1^ HAuCl_4_ solution suspended in (b) pure toluene and (c) toluene solution of 3 mg mL^−1^ Py during the extraction process. (d) Time‐dependent interfacial tension of toluene solution containing 10 mg mL^−1^ Py and 0.3 mg mL^−1^ AuCl_4_^−^ solution with different pH values. (e) The final interfacial tension and surface coverage as a function of pH. (f) Time‐dependent interfacial tension of toluene solution containing 3 mg mL^−1^ Py and AuCl_4_^−^ solution (pH 4) with different concentrations. (g) The final interfacial tension and surface coverage as a function of AuCl_4_^−^ concentration. (h) Time‐dependent interfacial tension of 0.1 mg mL^−1^ AuCl_4_^−^ solution (pH4) and toluene solution containing different concentrations of Py. (i) The final interfacial tension and surface coverage as a function of AuCl_4_^−^ concentration. Snapshots of the droplet before extraction and after wrinkles appeared are inserted (e, g, i). Scale bar, 2 mm.

As the PPy film is formed in situ at the liquid–liquid interface, the interfacial tension (IFT) can serve as an indicator for monitoring the redox reaction process. Typically, an aqueous droplet containing AuCl_4_^−^ ions is injected into an organic solvent (e.g., toluene) with dissolved pyrrole (Py) monomers. After aging, the droplet volume is extracted, during which wrinkling occurs due to the presence of the PPy film polymerized from Py monomers at the interface (Figure 2a). To validate this process, we injected an aqueous tetrachloroauric acid (HAuCl_4_) solution (0.8 mg mL^−1^) into toluene either in the absence or presence of Py molecules (Figure 2b,c; Video S1). In contrast to the smooth interface observed in pure toluene, pronounced wrinkles rapidly emerged on the droplet surface in the toluene solution containing 3 mg mL^−1^ Py monomers after compression, indicating extensive interfacial coverage by the formed PPy film. The interfacial film remains intact and robust throughout repeated injection‐extraction cycles, demonstrating its solid‐like character and inherent mechanical stiffness.

Since the pH of the solution significantly influences redox reactions [22], we investigated this effect by preparing a series of AuCl_4_^−^ solutions (0.3 mg mL^−1^) with pH values ranging from 0.94 to 13.22. Figure 2d presents the time‐dependent interfacial tension (IFT) trends of these aqueous solutions against toluene containing 10 mg mL^−1^ Py monomers. In all cases, the IFT reached equilibrium within ∼30 min, and this equilibration time shortened with increasing pH. For pH ≤ 7, the IFT stabilized at ∼21 mN m^−1^. In contrast, under alkaline conditions (pH > 7), the equilibrium IFT exhibited a further decrease with increasing pH. To correlate these IFT trends with film formation, we measured the surface coverage (SC) after aging the droplets for 1 h. The SC remained high (98%) in acidic media but dropped markedly when the solution pH exceeded 7 (Figure 2e). These results indicate that an acidic environment facilitates the interfacial redox reaction, thereby promoting robust film formation. Conversely, alkaline conditions hinder the process, due to the side reaction between the AuCl_4_^−^ ions and OH^−^ [23], leading to a significant reduction in both the final IFT and surface coverage. Based on these findings, the pH of the AuCl_4_^−^ solution used in this study was fixed at 4 unless otherwise specified.

To further investigate the influence of reactant concentrations on the interfacial film formation, we examined AuCl_4_^−^ solutions at concentrations ranging from 0 to 1.0 mg mL^−1^ in contact with toluene containing 3 mg mL^−1^ Py monomers. As shown in Figure 2f,g and Video S2, increasing the AuCl_4_^−^ concentration from 0 to 1.0 mg mL^−1^ led to only a modest IFT decrease from 35 to ∼27 mN m^−1^, indicating a limited effect of AuCl_4_^−^ on the water‐toluene interfacial tension. In contrast, the SC exhibited a pronounced dependence: a slight increase in AuCl_4_^−^ concentration resulted in a sharp rise in SC. This was clearly demonstrated by the increase in coverage from 0% to 100% as the AuCl_4_^−^ concentration was raised from 0 to 1.0 mg mL^−1^.

We similarly evaluated the effect of Py concentration by varying it from 0.1 to 50 mg mL^−1^ while maintaining the AuCl_4_^−^ concentration at 0.1 mg mL^−1^. In the absence of Py, the IFT remained constant at 35 mN m^−1^, indicating no significant interfacial activity (Figure 2h). At low Py concentrations (≤ 3 mg mL^−1^), the IFT fluctuated near 32 mN m^−1^, with SC stabilizing around 37%. When the Py concentration exceeded 3 mg mL^−1^, the IFT decreased noticeably, and the corresponding SC increased. However, the SC plateaued at ∼76% within the 20–50 mg mL^−1^ Py concentration range, suggesting interfacial saturation by the film under these conditions.

Furthermore, the film formation process directly governs the mechanical robustness of the resulting interfacial film. As shown in Figure S2, when the AuCl_4_^−^ concentration was increased to 5 mg mL^−1^ or higher, the interfacial film became remarkably stiff even at a low pyrrole concentration (0.5 mg mL^−1^), exhibiting sufficient mechanical strength to support the underlying organic phase upon vessel inversion. Remarkably, even after being punctured with a needle, the film retained its load‐bearing capacity without any leakage (Figure S3), indicating that its mechanical integrity remained essentially unchanged before and after repair.

### 2.3 Conductivity and Printability of the Liquid‐in‐Liquid 3D Printed Liquid Tubular Wires

We propose that the in situ formation of a PPy film at the water–oil interface can enhance its conductivity, despite the non‐conductive nature of the pristine Py monomers. To verify this, the electrical properties of the film were characterized using two‐point probe measurements in both direct current (DC) and alternating current (AC) modes (Figures S4 and S5). In DC mode, the time‐dependent current was measured under a constant voltage after 20 h to minimize interference with the film formation process. The current stabilized at ∼40 µA, which is significantly higher than that of the AuCl_4_^−^ solution (nA level) or a dichloromethane (DCM) solution containing Py monomers (nearly zero), indicating the relatively high conductivity of the interfacial PPy film (Figure S4a). Furthermore, when the electrodes were retracted from the interface, the current dropped abruptly to a nanoscale value (Figure S4b), confirming that the conductivity originates from the PPy film at the interface. To account for potential electrode polarization effects, impedance spectroscopy was performed under an AC voltage (Figure S5). The Nyquist plot of the AuCl_4_^−^ solution alone showed a prominent arc in the high‐frequency region (Figure S5a). In contrast, the charge transfer resistance (R_ct_) at the water–oil interface was substantially reduced in the presence of the PPy film and decreased further with increasing AuCl_4_^−^ concentration (Figure S5b). At an AuCl_4_^−^ concentration of 3 mg mL^−1^, the arc nearly disappeared, suggesting highly efficient electron transport across the film. These results collectively demonstrate a dramatic reduction in interfacial R_ct_ due to the in situ‐formed PPy film, reaffirming its high conductivity. It provides a foundation for developing liquid tubular wires from nonconductive molecular precursors.

**FIGURE 3:**
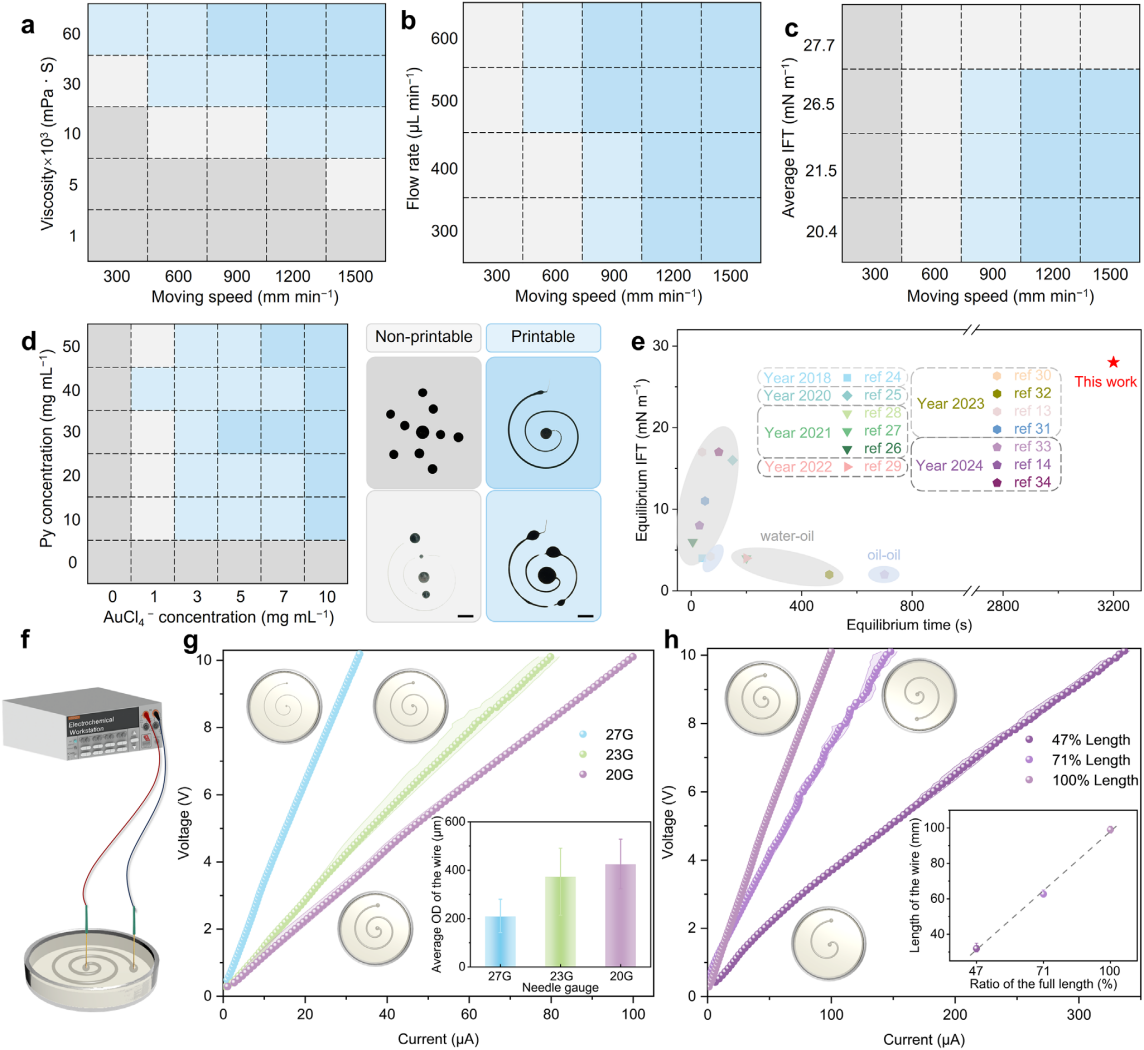
Liquid‐in‐liquid 3D printed liquid tubular wires and their conductivity. (a–c) Profiles of the printing window showing the mutual effects of parameters: needle moving speed, viscosity of the silicone oil (a), injection flow rate (b), and the IFT of two phases (c) on the printing. (d) Suitable concentration profile of AuCl_4_^−^ solution and Py monomers in silicone oil (60 000 mPa·S) for liquid‐in‐liquid 3D printing. Scale bar, 5 mm. (e) Comparison of the equilibrated IFT and equilibrium time for the liquid 3D printing enabled by this work and previous reports [13, 14, 24, 25, 26, 27, 28, 29, 30, 31, 32, 33, 34]. (f) Schematic of the experimental setup for measuring the conductivity of the liquid tubular wires. Profiles of the current of the circuit connected by the liquid tubular wires with (g) different diameters and (h) lengths as a function of the DC voltage. Insets are the actual outer diameters (ODs) and lengths of the printed liquid tubular wires, with their corresponding diagrammatic figures.

However, unlike surfactants that substantially reduce the IFT between aqueous and organic phases, neither Py monomers nor AuCl_4_^−^ ions exhibit interfacial activity, resulting in a high IFT that complicates the liquid‐in‐liquid 3D printing of such systems. To address this challenge, we systematically optimized printing parameters, including viscosity of the silicone oils, needle moving speed, ink flow rate, the IFT between the two phases, and reactant concentrations. We first investigated the interdependent effects of bath viscosity, needle moving speed, ink flow rate, and the IFT on printing performance (Figure 3a–c). Higher viscosity of the silicone oil, increased injection flow rate, lower IFT, and faster needle movement all favored the printing of continuous structures (Figures S6–S8). The key to successful printing lies in overcoming Plateau‐Rayleigh instabilities induced by high IFT. A high moving speed generates greater inertial forces to counteract these instabilities, while an elevated flow rate supplies more reactants to the system, facilitating interfacial film formation and thus reducing IFT. Meanwhile, high‐viscosity silicone oils enhance inertial stabilization and extend the time available for interfacial reactions, further contributing to IFT reduction. Collectively, these parameters directly or indirectly mitigate the impact of Plateau‐Rayleigh instabilities during printing. Based on these insights, we establish a quantified printing window with optimized parameters, providing a practical reference for 3D printing in high‐IFT liquid systems. Subsequently, we chose the viscosity (60 000 mPa·S), the speed (1200 mm min^−1^), flow rate (500 µL min^−1^), and needle diameter (0.91 mm) as optimal conditions to systematically evaluate the effect of reactant concentrations. As summarized in Figure 3d and Figure S9, continuous and intact spiral structures could be printed without breakpoints only within specific concentration ranges of Py and AuCl_4_^−^. Outside these ranges, the printed structures rapidly fragmented into disconnected segments and spherical droplets due to Plateau‐Rayleigh instabilities (Figure S9). According to prior studies on liquid‐in‐liquid 3D printing [13, 14, 24, 25, 26, 27, 28, 29, 30, 31, 32, 33, 34], successful printing typically requires both optimized process parameters and a rapid reduction in IFT between the ink and the matrix to suppress Plateau‐Rayleigh instabilities. Consequently, printable liquid–liquid systems generally exhibit either low equilibrium IFT (below 20 mN m^−1^) or short IFT equilibration times (below 700 s), particularly in systems intended for conductive applications (Figure 3e). In contrast, we achieved printing continuous liquid tubular wires from nonconductive molecular precursors under relatively high IFT (∼28 mN m^−1^) and considerably long equilibration time (∼3200 s) by employing this printing strategy (Figure 3e). This demonstrates that the mechanically robust PPy film formed via interfacial redox reaction is sufficiently stiff to stabilize the printed liquid structure against deformation.

Our method enables the 3D printing of both in‐plane and out‐of‐plane structures (Figure S10 and Video S3), showing promise for applications in underwater electronic devices. Conductive solid wires (e.g., metal wires) obey Ohm's law, a defining characteristic of such conductors. To evaluate whether the printed liquid tubular wires exhibit Ohmic behavior, we fabricated and characterized wires with varying diameters and lengths. As illustrated in Figure 3f, the current was measured under applied DC voltages ranging from 0 to 10 V. The liquid tubular wires of different diameters were printed using 20‐gauge (20G), 23‐gauge (23G), and 27‐gauge (27G) needles under consistent printing parameters (Figure S11). The resulting average diameters were 424, 372, and 208 µm, respectively (inset, Figure 3g). As shown in Figure 3g, at the measured range of voltage, the current increased linearly with voltage for all liquid tubular wires, regardless of diameter, indicating Ohmic behavior. However, the slopes of these lines, corresponding to the resistance of the liquid tubular wire (R*_l_*), varied significantly. After fitting the lines, R*_l_* decreased with increasing wire diameter, from 303 kΩ (27G) to 133 kΩ (23G), and finally to 99.7 kΩ (20G) (Figure S12), suggesting that R*_l_* is inversely proportional to the cross‐sectional area. Similarly, we also printed the liquid tubular wires of different lengths using a 20G needle (Figure S13), with actual lengths measuring 32, 63, and 99 mm, corresponding to 47%, 71%, and 100% of the full spiral design, respectively (inset, Figure 3h). Here, the length ratio is the theoretically printed length to the full length of the spiral. All current–voltage curves remained linear (Figure 3h), confirming adherence to Ohm's law. The fitted resistance was proportional to the length of the printed liquid tubular wire, decreasing from 99.7 to 29 kΩ as the wire length was reduced from 100% to 47% (Figure S14). These results demonstrate that the resistance R*_l_* follows the relationship R = ρL/S (where ρ, L, and S represent density, length, and cross‐sectional area of the conductor, respectively), confirming that the liquid tubular wires exhibit solid‐like electrical characteristics.

### 2.4 Liquid Tubular Wires as Reconfigurable Conductors

**FIGURE 4:**
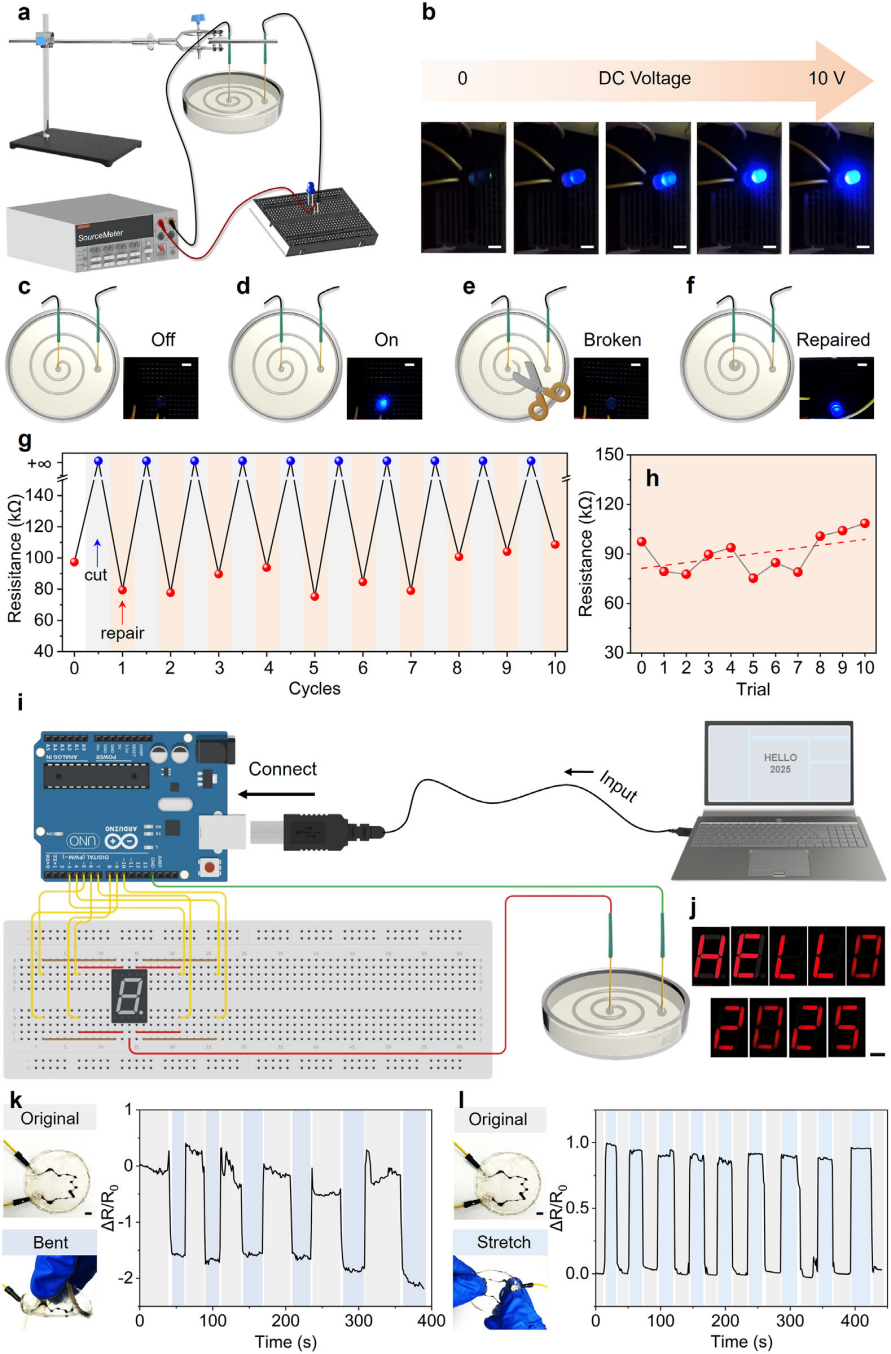
Electrical applications of the printed liquid tubular wire. (a) Schematic representation of the simple circuit connected by the liquid tubular wire. (b) Photographic images showing the change in brightness of an LED with the increase in DC voltage. Snapshots of the LED when the circuit is (c) turned off and (d) on, and the circuit with (e) the broken and (f) the repaired liquid tubular wire. (g) Profile of resistance changes between the disconnected and repaired circuit during cut‐repair cycles. (h) Tendency of the resistance of the repaired wire during 10 trials. (i) Schematic illustrating the circuit diagram of the information transmission enabled by the liquid tubular wire. (j) Photographic images of the letter and number displayed on the 7‐segment display indicator. (k) Real‐time signal response before and after bending the home‐made sensor prototype, with the images showing the original/bent‐state prototype (left). (l) Real‐time signal response before and after stretching the home‐made sensor prototype, with the images showing the original/stretched‐state prototype (left). Stretched length is 115% of the original length. R_0_ is the resistance of its original state, while ΔR is the change in resistance when bending or stretching the sensor. Scale bars, 5 mm.

We first evaluated the thermal stability of the printed tubular wire as a conductor. As the temperature increased from 20°C to 100°C, R*_l_* exhibited only a slight decrease, indicating satisfactory thermal stability of its electrical performance (Figure S15). Having established that the printed liquid tubular wires exhibit key electrical characteristics of solid‐state conductors, they demonstrate considerable potential for use as functional wiring in electronic applications. To illustrate this capability, we constructed a DC circuit comprising a power supply and an LED connected by a printed liquid tubular wire, as schematically shown in Figure 4a. When the DC voltage was increased from 0 to 10 V, the LED gradually brightened in response (Figure 4b; Video S4). Benefiting from their intrinsic fluidity, the liquid tubular wires possess a distinctive ability to self‐reconfigure, which is an advantage not afforded by conventional solid‐state electronics. To evaluate this reconfigurability, we performed a series of sequential experiments (Figure 4c–f). Initially, a DC circuit was connected by inserting two Pt wires into opposite ends of the liquid tubular wire; the LED remained off when the switch was open (Figure 4c; Figure S16 and Video S4). Upon closing the switch and applying a constant voltage of 4 V, the LED illuminated brightly (Figure 4d, Figure S16, and Video S4), confirming that a complete current loop had been established through the liquid tubular wire. The LED immediately turned off when the wire was severed (Figure 4e; Figure S16 and Video S5). However, after injecting additional AuCl_4_^−^ solution into the gap at the break, the LED relit (Figure 4f; Figure S16), demonstrating successful restoration of the broken loop via the inherent reconfigurability of the liquid tubular wire. The electrical stability of the liquid tubular wire before and after repair was evaluated through successive cut‐repair cycles (Figure 4g,h; Figures S17 and S18). The resistance fluctuated around 90 kΩ during the first eight cycles and began to increase slightly only after the eighth repair, indicating robust electrical recoverability upon repeated reconfiguration.

Furthermore, to explore the potential of these liquid tubular wires as solid‐like conductors in practical applications, we demonstrated their capability for information transmission. An integrated circuit was assembled using an Arduino board, a breadboard, and a 7‐segment display indicator, interconnected via the printed liquid tubular wire (Figure S19). As illustrated in Figure 4i, coded information comprising letters and numbers was input to the Arduino, which then transmitted corresponding signals to the display. We programmed the system to output the message “HELLO 2025”. Upon switching on the circuit, the display sequentially showed the characters “H”, “E”, “L”, “L”, “O”, “2”, “0”, “2”, and “5”, accurately reproducing the intended message (Figure 4j; Video S6). This experiment confirms that the liquid tubular wires can reliably transmit digital information such as numbers, letters, and encoded sentences, highlighting their great potential for use in informatics applications, including Morse code transmission and other signal‐based communication systems.

Additionally, we have opened up more possibilities for printed liquid tubular wires. To further reduce the IFT of the system, we added dodecylbenzene sulfonic acid (DSA), acting as the surfactant and dopant, into the aqueous solution. This enabled the smooth printing of more complex geometries, including heart‐, rhombus‐, and butterfly‐shaped patterns, as well as 3D circular rings (Figure S20). We then fabricated a proof‐of‐concept sensor by printing the wire into a polymerizable Sylgard 184 (Dow Corning) bath, followed by curing for two days. The encapsulated wire exhibited excellent mechanical adaptability, allowing repeated bending and stretching without failure (Figure S21a). Electrical characterization confirmed that the wire 100% obeyed Ohm's Law, as the current proportionally increases with the voltage (Figure S21b). It revealed that the wire has effectively transitioned to a solid‐state conductor. As shown in Figure 4k,l, the resistance was reduced responsively when bending the prototype but rebounded to the original value after recovery, while the resistance increased rapidly when stretching the prototype but returned to normal after recovery. These responsive resistance changes remained consistent over multiple cycles, demonstrating the sensor's durability and signal stability. These results highlight the potential of our liquid tubular wires as building blocks for wearable sensors and other adaptive electronic devices.

## 3 Conclusion

In summary, we have demonstrated the construction of reconfigurable liquid tubular wires via liquid‐in‐liquid 3D printing using initially non‐conductive molecular precursors. Utilizing the interfacial redox reaction between AuCl_4_^−^ and Py at the water–oil interface, a mechanically stiff and conductive PPy film embedded with AuNPs is formed in situ, enabling the shaping and stabilization of all‐liquid structures. The film formation process depends critically on reactant concentration and solution pH, with acidic conditions and higher precursor concentrations favoring robust film growth. By systematically optimizing printing parameters, we achieve stable printing despite high IFT and long equilibration time, and present a printing window for such high‐IFT systems. The resulting liquid tubular wires exhibit solid‐like conductive behavior, functioning as real electrical components that can be connected, severed, and repaired on demand. Moreover, we demonstrate for the first time the ability of such liquid tubular wires to transmit digital information, and their utility as proof‐of‐concept sensors, capable of detecting distinct electrical signals in response to external mechanical forces or other stimuli, highlighting their potential for soft electronics and wearable sensors. This work establishes a pioneering approach to constructing conductive all‐liquid electronics from non‐conductive materials, opening new pathways toward adaptive and reconfigurable electronic systems for next‐generation applications.

## 4 Experimental Section

### 4.1 Materials

Tetrachloroauric acid (HAuCl_4_), NaOH (97%), ethanol (≥99.5%), hydrochloric acid (36%), dodecylbenzene sulfonic acid (DSA) (≥90%), and silicone oils (η_e_ ∼1000, 10 000, 30 000, 60 000 mPa·s) were purchased from Aladdin. Pyrrole (99%) and silicone oil (η_e_ ∼5000 mPa·s) were provided by Macklin. Organic solvents, including toluene (99.5%) and dichloromethane (DCM, 99.5%), were obtained from Sigma–Aldrich. Sylgard 184 silicone elastomer base and agent were supplied by Dow Corning. All chemicals were used without further purification unless specified. Deionized water (DIW) was obtained from a Milli‐Q system.

### 4.2 Characterizations

Morphologies and chemical compositions of the formed interfacial films were tested by scanning electron microscopy (SEM, operated at 20.0 kV, JEOL JSM‐IT500) and X‐ray photoelectron spectroscopy (XPS, PHI Model 5802). The binding energy used the C 1s peak at 284.8 eV as a reference. The interfacial tension (γ) was analyzed by a multifunctional tensiometer (Dataphysics Instruments OCA15EC, Germany) using the pendent‐drop method. The deformation and wrinkle behavior were recorded as figures or videos with a digital camera. The pH values of aqueous AuCl_4_^−^ were measured by the pH meter (FiveEasy Plus FP20, Mettler Toledo, Switzerland). The conductivity measurements were carried out by Gamry Instruments (Interface 1010E, USA). The commercial Arduino UNO and power supply (Thurlby‐Thandar Instruments Ltd., UK) were used to demonstrate some applications of the printed all‐liquid devices.

### 4.3 Sample Preparation for Surface Measurements

The film was deposited onto a silicon substrate using a custom‐designed setup connected to a syringe pump. Prior to the introduction of liquid phases, a clean silicon wafer was positioned on the sample stage within the device, fully immersed in the dichloromethane (DCM) phase. A DCM solution containing 1 mg mL^−1^ Py monomers was first injected until the silicon wafer was completely covered. Subsequently, an aqueous solution of AuCl_4_^−^ (0.5 mg mL^−1^, pH 4) was gently layered on top of the DCM phase. Following a 24‐h interfacial reaction, the DCM phase was slowly drained through a bottom outlet via the syringe pump, allowing the formed interfacial film to gradually deposit onto the silicon substrate. The resulting film‐on‐wafer sample was then rinsed five times with ethanol and deionized water, and finally dried in an oven overnight before being subjected to surface characterization, including SEM and XPS.

### 4.4 Conductivity Measurements

The electrical conductivity of the interfacial film was evaluated using both DC and AC measurement modes. In a typical procedure, a DCM solution containing Py monomers was first introduced into a vessel. Two Pt wires (0.5 mm in diameter) were then vertically inserted into the DCM phase as electrodes, with a fixed inter‐electrode distance of 6 mm. An equivalent volume of AuCl_4_^−^ solution was carefully layered on top of the DCM phase. To minimize interference from the applied voltage on the interfacial redox reaction, all electrical measurements, including chronoamperometry (DC mode) and potentiostatic electrochemical impedance spectroscopy (EIS, AC mode, frequency range from 2 MHz to 0.1 Hz), were initiated after a 20‐h reaction period. For conductivity measurements of the printed liquid tubular wires, two Pt wires (0.5 mm diameter) were inserted into opposite ends of a wire printed 30 min prior. The electrical characterization was then performed using linear sweep voltammetry.

### 4.5 Liquid‐in‐Liquid 3D Printing of the Liquid Tubular Wire

To enable stable 3D printing of the liquid tubular wires, a high‐viscosity silicone oil (60 000 mPa·s) was employed as the matrix using the Snapmaker 3D printer (Snapmaker Inc., China). In a standard procedure, an aqueous AuCl_4_^−^ solution (10 mg mL^−1^) was extruded through a 20‐gauge stainless‐steel needle into the silicone oil containing 20 mg mL^−1^ Py monomers. The aqueous ink flow rate and the needle moving speed were set at 500 µL min^−1^ and 1200 mm min^−1^, respectively. Unless otherwise stated, these parameters were kept constant for all printed structures.

### 4.6 Fabrication of the Liquid‐Tubular‐Wire Sensor

To explore more possibilities of application, an aqueous mixture of AuCl_4_^−^ solution (20 mg mL^−1^) and DSA (10 mg mL^−1^) was extruded through a 20‐gauge stainless‐steel needle into a curable Sylgard 184 bath containing 20 mg mL^−1^ Py monomers, at a needle moving speed of 1200 mm min^−1^ and an injection flow rate of 500 µL min^−1^. The printed wire and the bath were kept still for curing for 2 days. Finally, the sensor was connected to a circuit to measure the current under a constant DC voltage of 1 V.

## Funding

The authors acknowledge financial support from the Research Grants Council of Hong Kong (Project No.21304421, Y.C.), the National Natural Science Foundation of China (Project No. 22003053, Y.C.), the Natural Science Foundation of Guangdong Province, China (Project No. 2023A1515011457, Y.C.), CityU Strategic Interdisciplinary Research Grant (Project No. 2020SIRG035, Y.C.).

## Conflicts of Interest

The authors declare no conflicts of interest.

## Supporting information




**Supporting File**: advs74752‐sup‐0001‐SuppMat.docx.

**Supporting File**: advs74752‐sup‐0002‐VideoS1.mp4.

**Supporting File**: advs74752‐sup‐0003‐VideoS2.mp4.

**Supporting File**: advs74752‐sup‐0004‐VideoS3.mp4.

**Supporting File**: advs74752‐sup‐0005‐VideoS4.mp4.

**Supporting File**: advs74752‐sup‐0006‐VideoS5.mp4.

**Supporting File**: advs74752‐sup‐0007‐VideoS6.mp4.

## Data Availability

The data that support the findings of this study are available from the corresponding author upon reasonable request.
