# Supplementary material for: Solid‐Like yet Reconfigurable 3D‐Printed Liquid Tubular Wires From Nonconductive Molecules
Source: Adv Sci (Weinh). 2026 Mar 9;13(27):e24287. doi: 10.1002/advs.202524287 (PMC13170235; doi:10.1002/advs.202524287)
Supplement: Supplementary file 1 — Supporting File: advs74752‐sup‐0001‐SuppMat.docx. [file ADVS-13-e24287-s002.docx]

Supporting Information

Solid-like yet Reconfigurable 3D-Printed Liquid Tubular Wires from Nonconductive Molecules

Yuchen Fu, Weixi Wu, Wei Chen, Jiangang Zhang, Biyu Jin, Sai Zhao, and Yu Chai*

Y. Fu, W. Wu, W. Chen, J. Zhang, S. Zhao, Y. Chai

Department of Physics, City University of Hong Kong, 83 Tat Chee Avenue, Kowloon, Hong Kong SAR, China

City University of Hong Kong Shenzhen Research Institute, 8 Yuexing 1st Road, Gaoxin District, Shenzhen, China

E-mail: yuchai@cityu.edu.hk

B. Jin

Materials Science and Engineering Program and Texas Materials Institute, The University of Texas at Austin, Austin, Texas, 78712 USA

**The Supplementary Information includes:**

Supplementary Text

Figures S1-S21

Table S1

Videos S1-S6

**Captions for Videos**

**Video S1.**

Evolution of pendent droplet with or without interfacial redox reactions.

**Video S2.**

Evolution of pendent droplet that reflects the effects of factors on the interfacial redox reactions.

**Video S3.**

Out-of-plane liquid-in-liquid 3D printing.

**Video S4.**

Tunable brightness of the LED bulb in the dark under different voltages of the circuit connected by the printed liquid wire.

**Video S5.**

Reconfigurability of the printed liquid wire.

**Video S6.**

Information transmission that is achieved by the liquid wire.

**

**

**Figure S1.** XPS spectrum of the prepared interfacial film.

**Table S1** Binding energies and surface elemental concentrations of the compositions of the interfacial film.

| **Compositions** | | **Binding Energy (eV)** | **Atomic %** |
| --- | --- | --- | --- |
| **Full Spectrum** | Au | 87.82 | 0.17 |
|  | C | 285.18 | 69.22 |
|  | N | 399.89 | 14.13 |
|  | O | 531.82 | 16.48 |
| **N 1s** | −N− | 399.43 | 84.28 |
|  | =N− | 398.02 | 15.72 |
| **C 1s** | C-C | 284.80 | 69.57 |
|  | C=N | 287.50 | 30.43 |
| **Au 4f** | Au^0^ 4f_7/2_ | 83.95 | 41.36 |
|  | Au^3+^ 4f_7/2_ | 86.60 | 4.90 |
|  | Au^0^ 4f_5/2_ | 87.57 | 44.50 |
|  | Au^3+^ 4f_5/2_ | 90.55 | 9.23 |

**
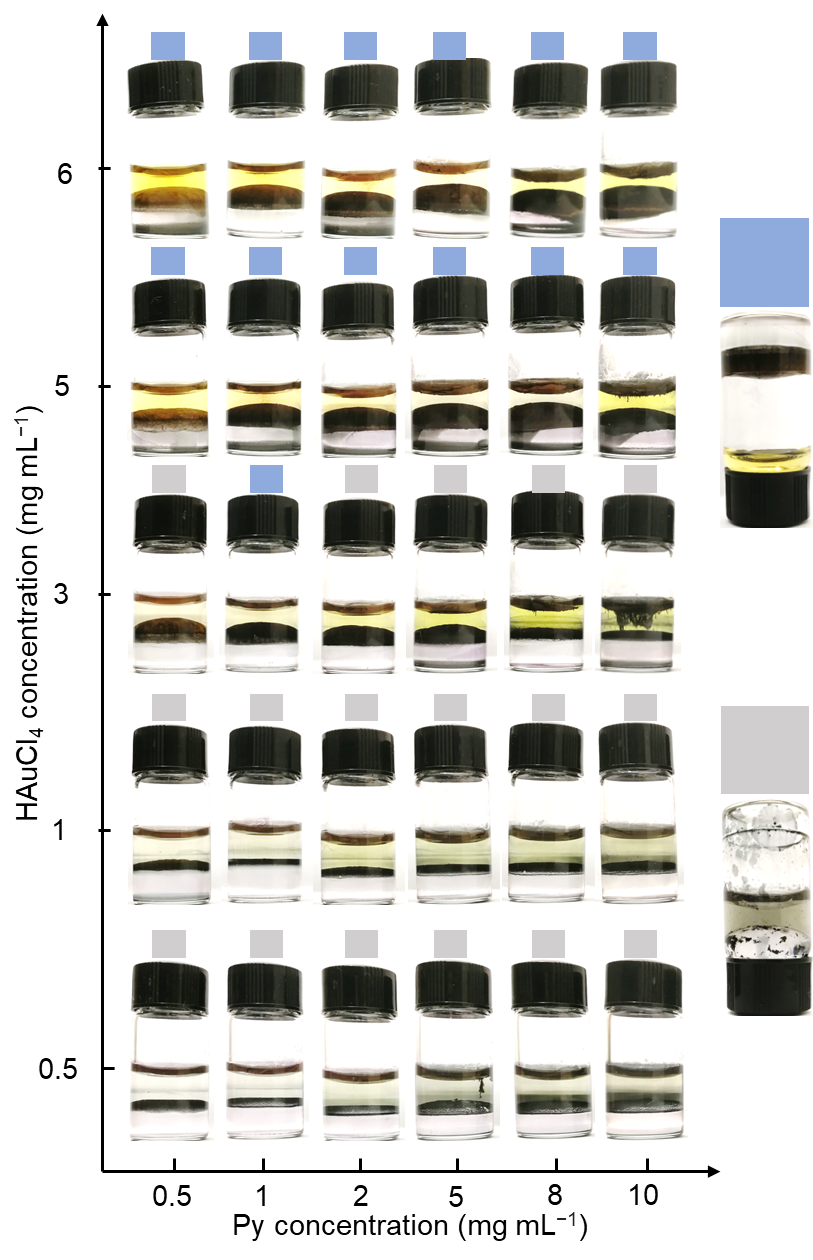
**

**Figure S2.** Snapshots of the vessels that were inverted and re-upright to show the integrity of the film formed at the interface of the aqueous solution containing different HAuCl_4_ concentrations from 0.5 mg mL^−1^ to 6 mg mL^−1^ and DCM containing different Py concentrations from 0.5 mg mL^−1^ to 10 mg mL^−1^ after 2 days. Bule square means the film is intact while the gray square means the film is broken.

**
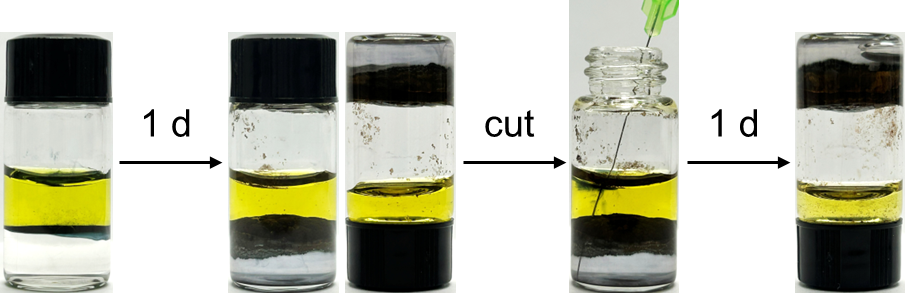
**

**Figure S3.** Snapshots of the vessels containing 10 mg mL^−1^ HAuCl_4_ and 2 mg mL^−1^ Py in DCM to show the mechanical strength of the film before and after repair.

**
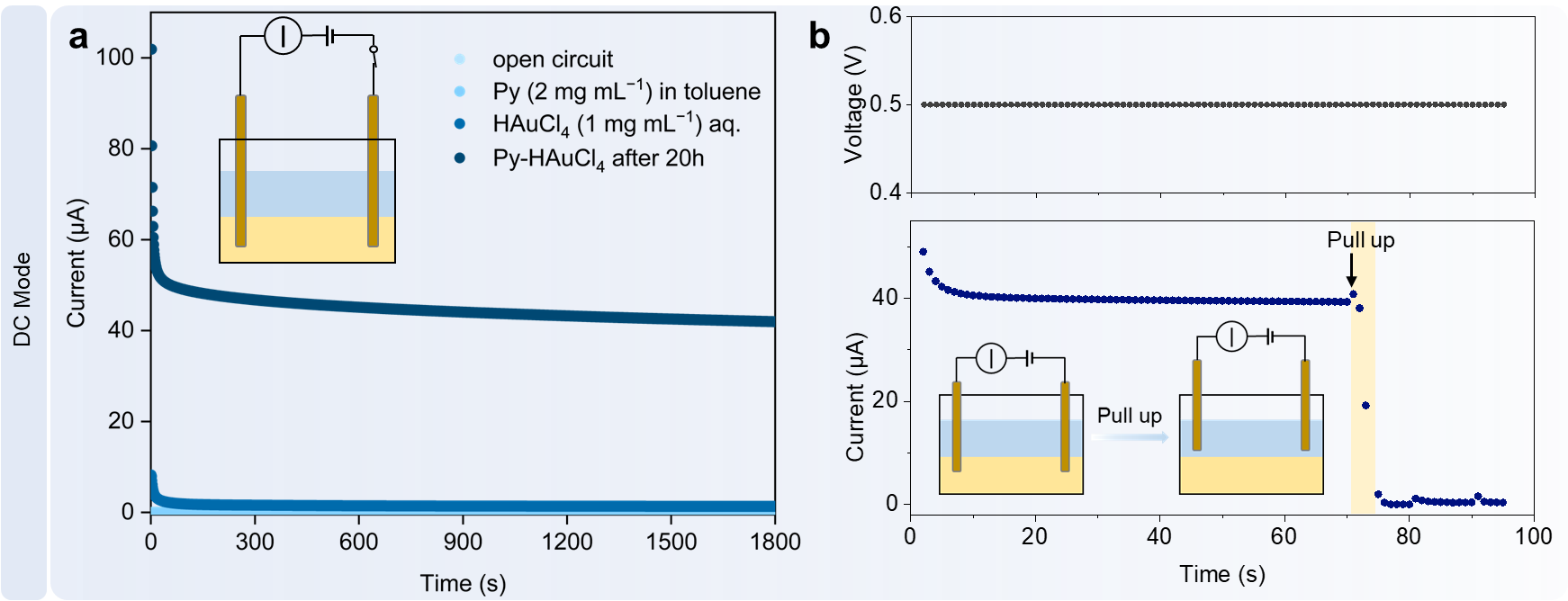
**

**Figure S4.** (a) Time-dependent currents of the DC circuit connected by the toluene solution containing 2 mg mL^−1^ Py monomers, aqueous HAuCl_4_ solution (1 mg mL^−1^), and the interface of toluene solution containing 2 mg mL^−1^ Py monomers and 1 mg mL^−1^ HAuCl_4_ solution after 20 hrs respectively, under a constant DC voltage of 0.5 V. (b) Changes in the current when pulling up the electrodes away from the film-formed interface under a constant DC voltage of 0.5 V. Experimental setup was inserted.

**
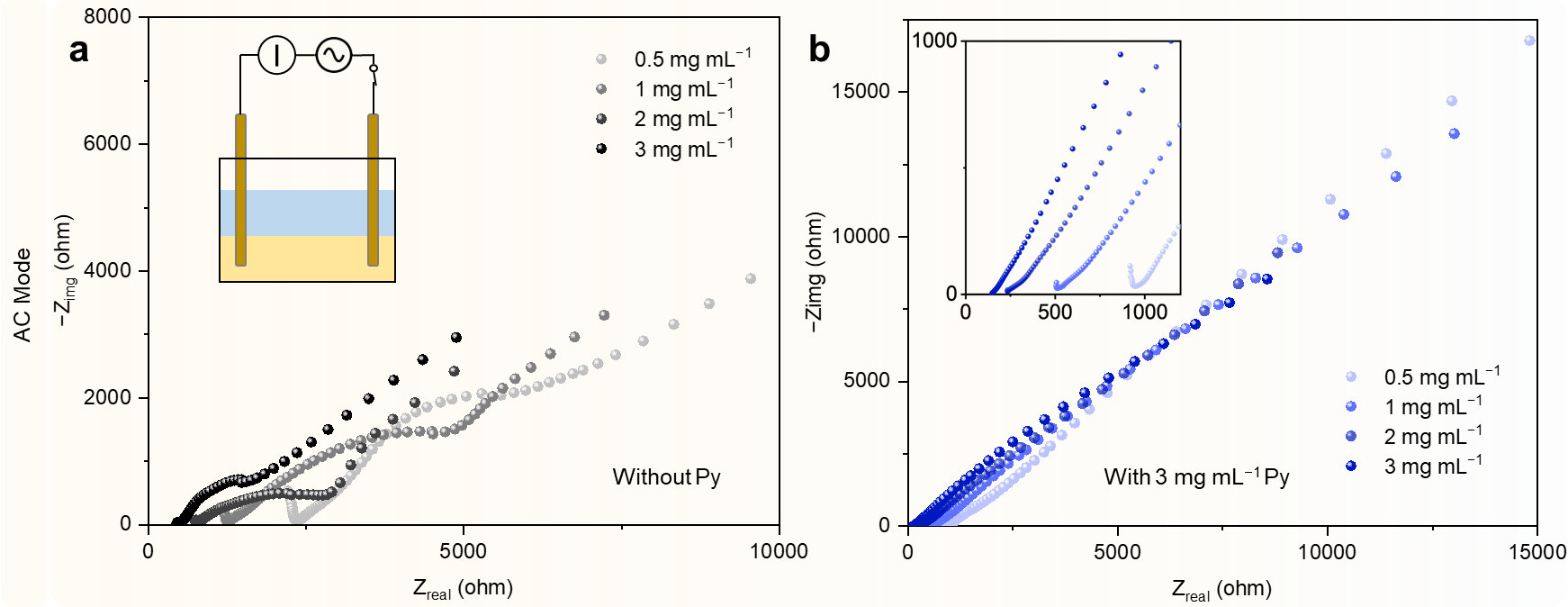
**

**Figure S5.** Nyquist plots measuring frequency from 2 MHz to 0.1 Hz for the interface of the aqueous AuCl_4_^−^ solution with different concentrations (0.5 mg mL^−1^ ~ 3 mg mL^−1^) and toluene without (a) or with 3 mg mL^−1^ Py monomers (b) after contacting 20 hrs. Experimental setup was inserted in (a) and the magnified view of the high-frequency region of impedance spectra of (b) was inserted in (b).

**
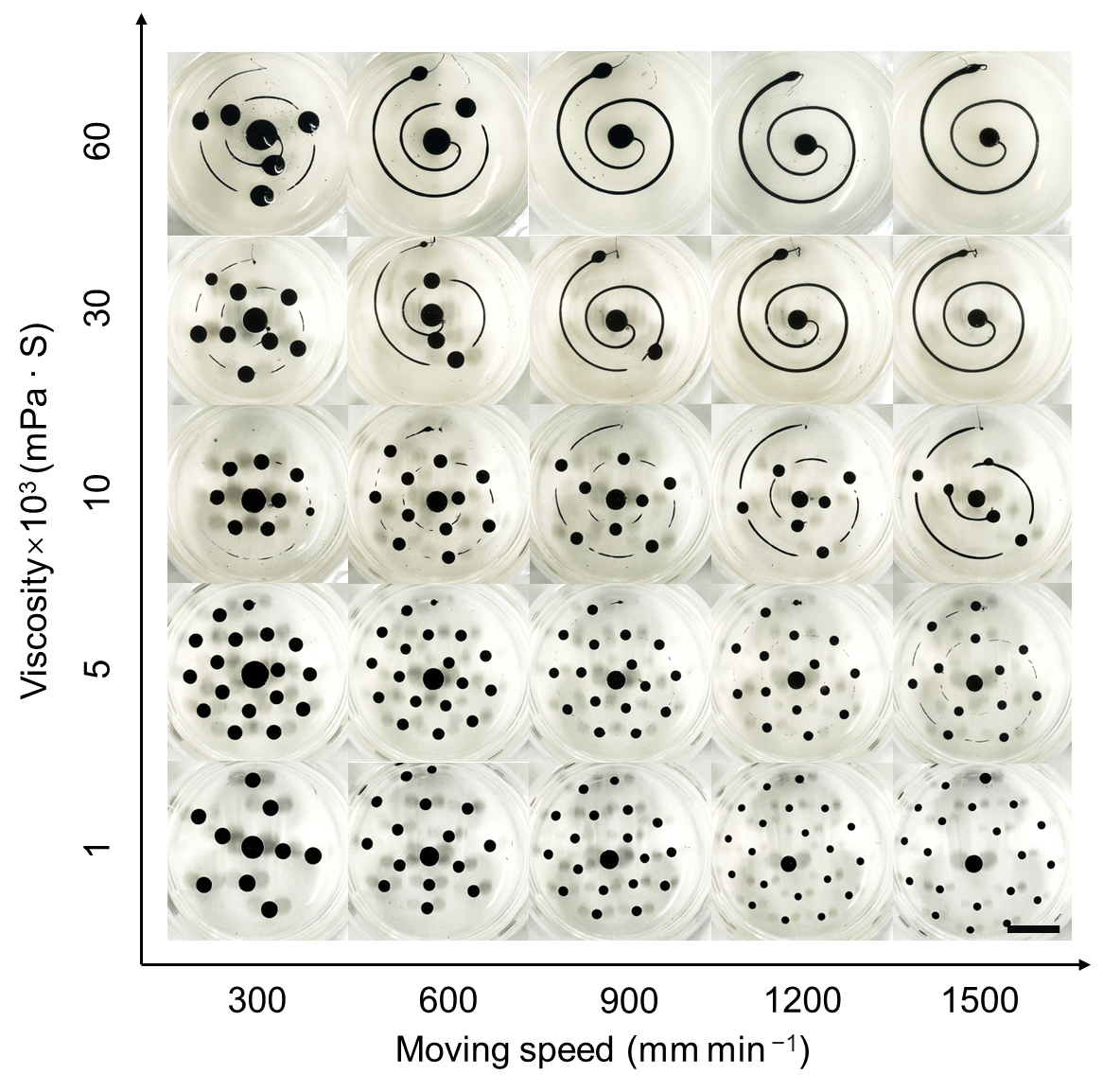
**

**Figure S6.** Effect of the viscosity of the silicone oil on the 3D printing. Photographic images of the structures printed by the AuCl_4_^−^ solution of 10 mg mL^−1^ into the silicone oil of 20 mg mL^−1^ Py molecules using a 20-gauge needle. The flow rate of the ink was 500 μL min^−1^, and the moving speed of needle was in the range of 300 ~ 1500 mm min^−1^. Scale bar, 1cm.

**
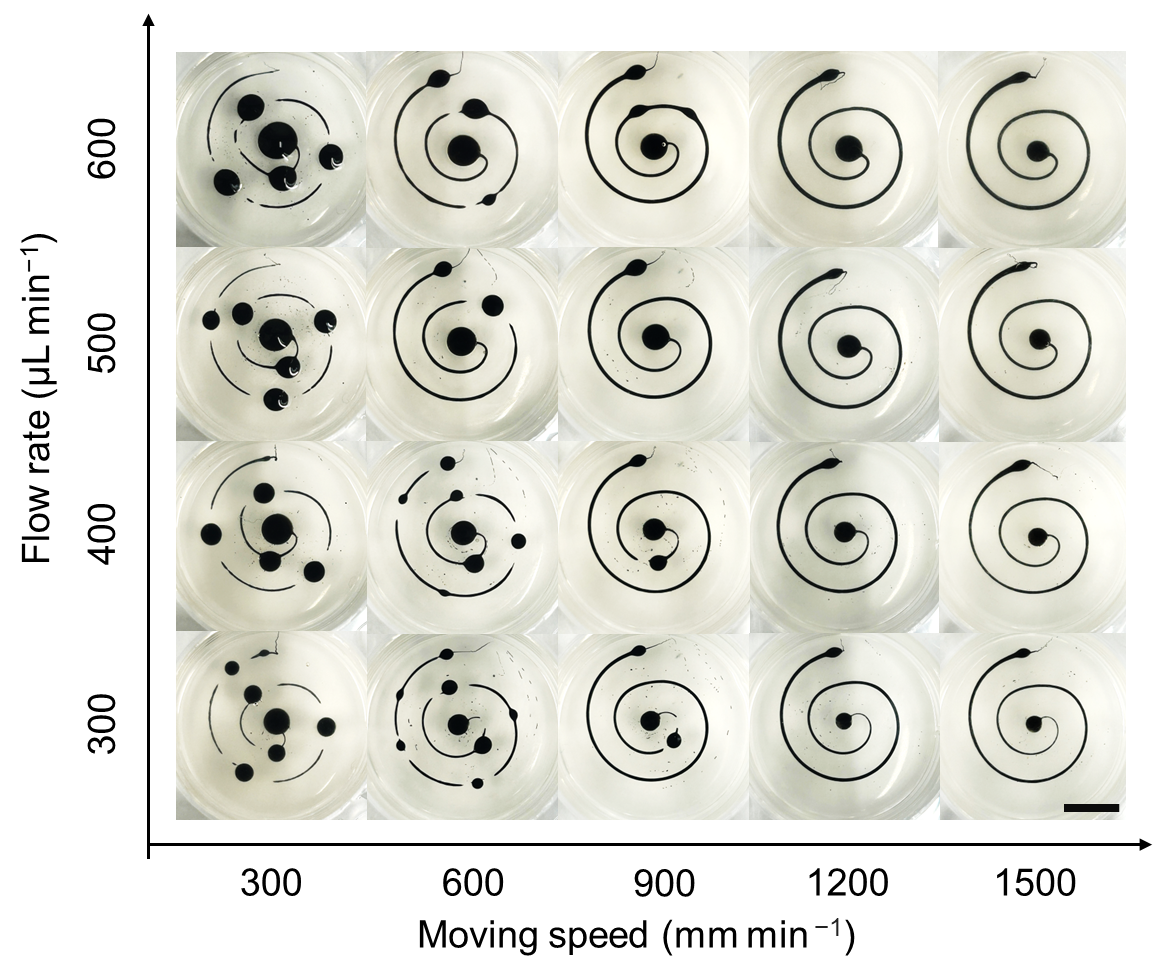
**

**Figure S7.** Effect of the flow rate on the 3D printing. Photographic images of structures printed by 10 mg mL^−1^ AuCl_4_^−^ solution into the silicone oil (60k mPa·S) of 20 mg mL^−1^ Py molecules at a flow rate from 300 μL min^−1^ to 600 μL min^−1^ using a 20-gauge needle. The moving speed of needle was in the range of 300 ~ 1500 mm min^−1^. Scale bar, 1cm.

**
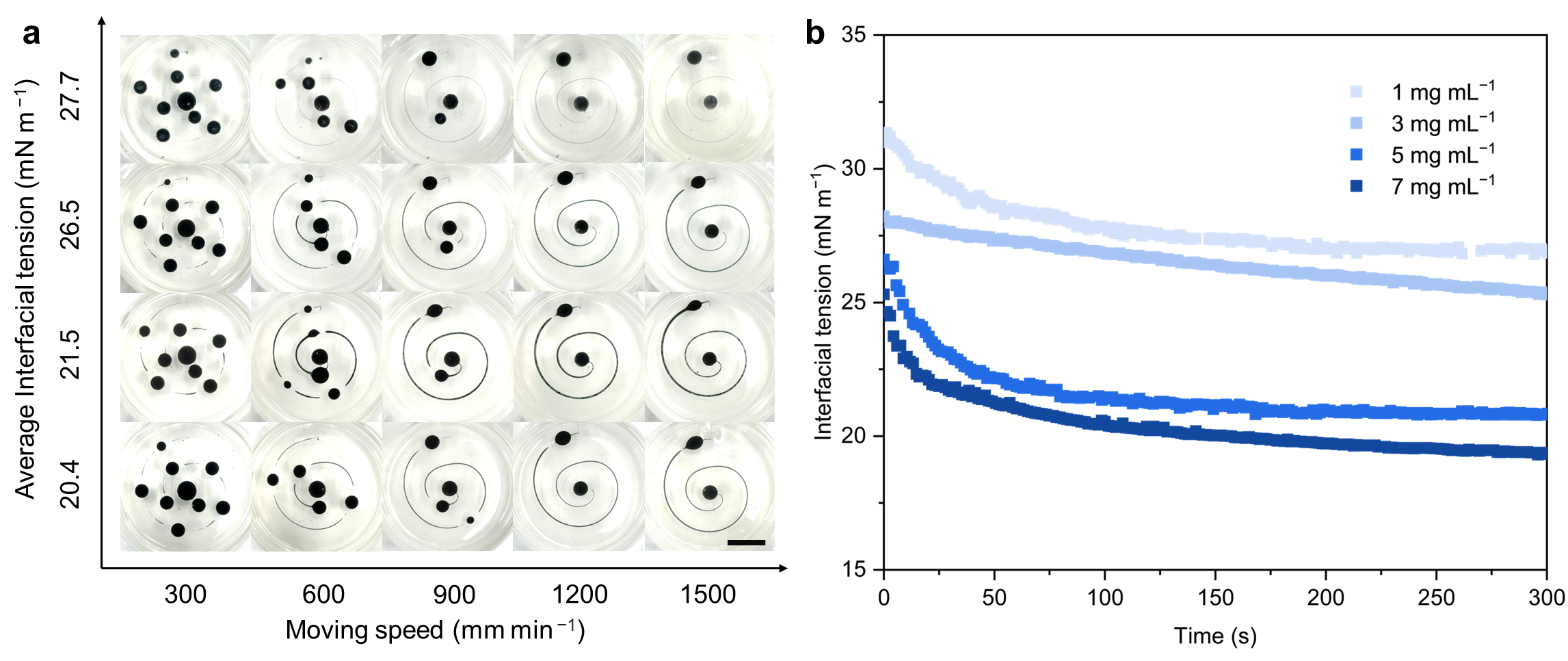
**

**Figure S8.** Effect of the IFT of water-oil phases on the 3D printing. (a) Photographic images of structures printed by differentAuCl_4_^−^ solutions into the silicone oil (60k mPa·S) of 10 mg mL^−1^ Py molecules at a moving speed of 300 ~ 1500 mm min^−1^ using a 20-gauge needle. The flow rate of the ink was 500 μL min^−1^. (b) Profile of IFT of the AuCl_4_^−^ solution with different concentrations (1, 3, 5, 7 mg mL^−1^) and 10 mg mL^−1^ Py in toluene. The average IFT was calculated using the data from (b) within 300 s. Scale bar, 1cm.

**
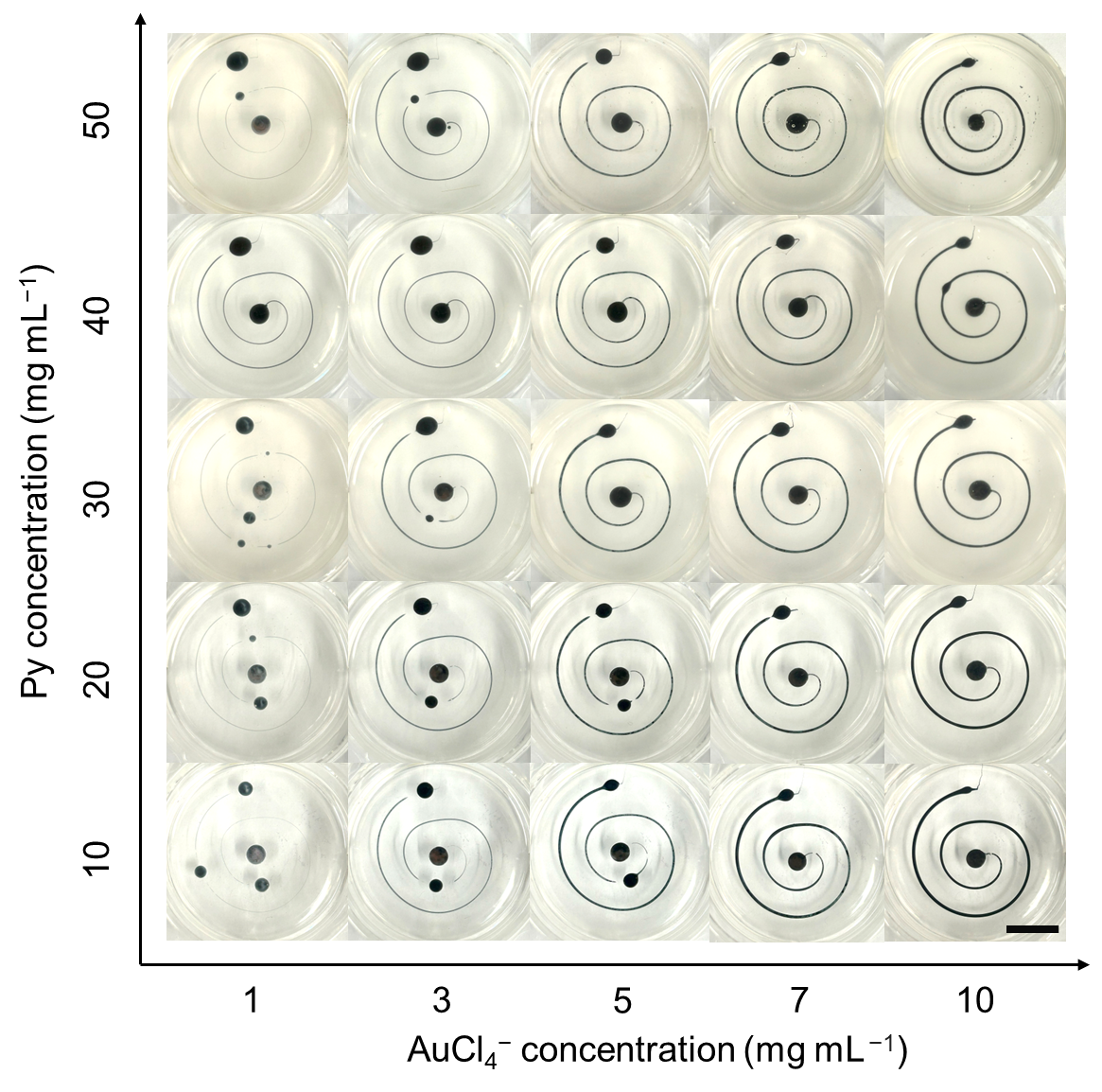
**

**Figure S9.** Parameter domain for the 3D printing based on the varied concentrations of the AuCl_4_^−^ ions (1 ~ 10 mg mL^−1^) and Py monomers (10 ~ 50 mg mL^−1^). The spiral was printed into the silicone oil (60k mPa·S) at a constant flow rate of 500 μL min^−1^ and moving speed of 1200 mm min^−1^ using a 20-gauge needle. Scale bar, 1 cm.

**
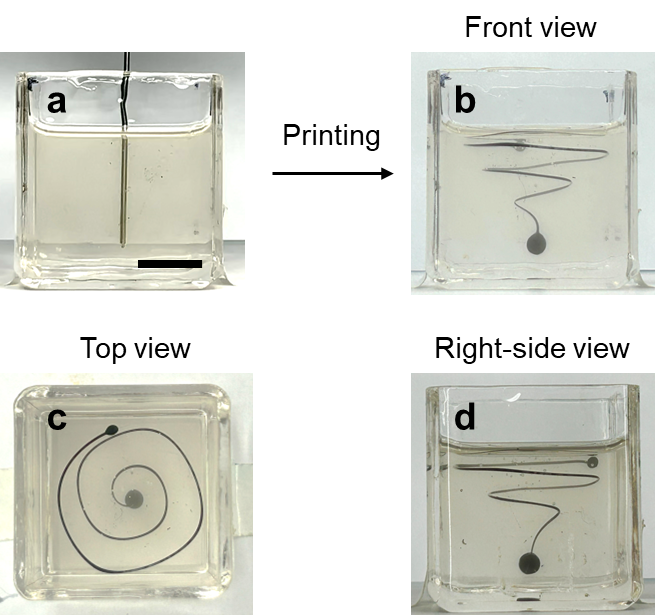
**

**Figure S10.** Demonstrations for the out-of-plane 3D printing. Snapshots of the process of the printing (a, b) and the final structure from the front view (b), top view (c) and right-side view (d). Scale bar, 1 cm.

**
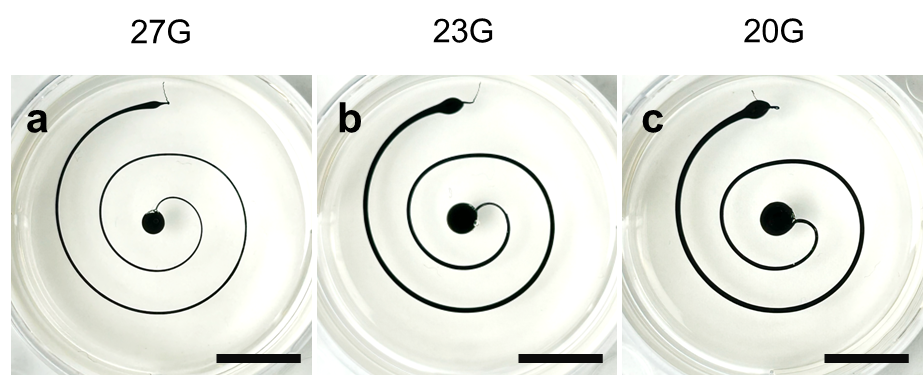
**

**Figure S11.** Liquid wires printed by the stainless-steel needles with different gauges. Photographic images of the liquid wires printed by the 27-gauge (a), 23-gauge (b), and 20-gauge (c) needles, respectively. Scale bar, 1 cm.

**
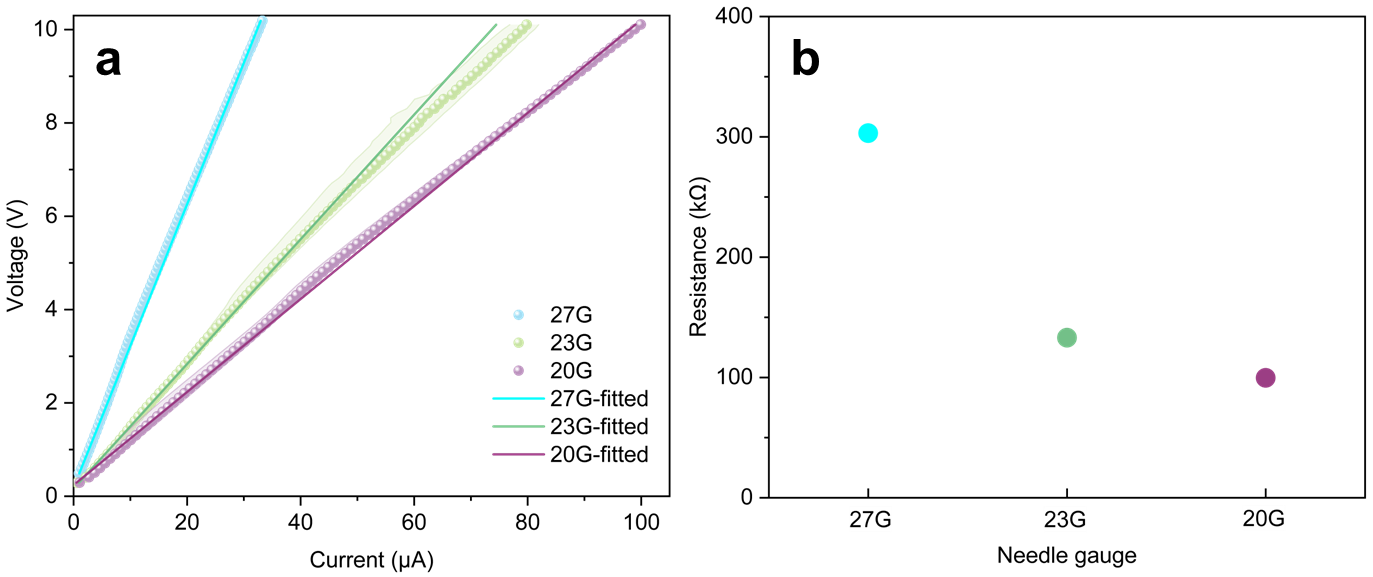
**

**Figure S12.** Conductivity of the liquid wires printed by different needles. (a) Plot of the current of the circuit connected by the liquid wire printed by needles with varied diameters as a function of DC voltages and the fitted curves. (b) The calculated resistance of the liquid wire obtained from (a).

**
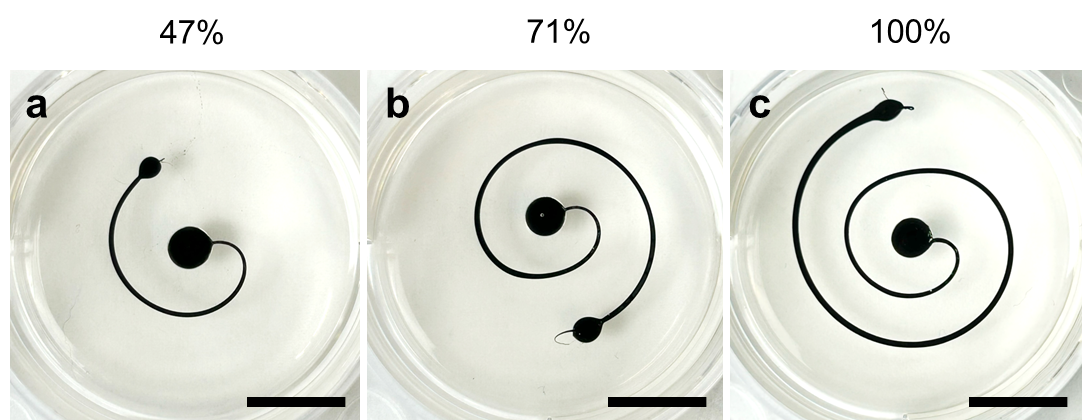
**

**Figure S13.** Liquid wires with different lengths printed by the 20-gauge needle. Photographic images of the printed liquid wires with 47% (a), 71% (b), and 100% (c) length of the wire, respectively. Scale bar, 1 cm.

**
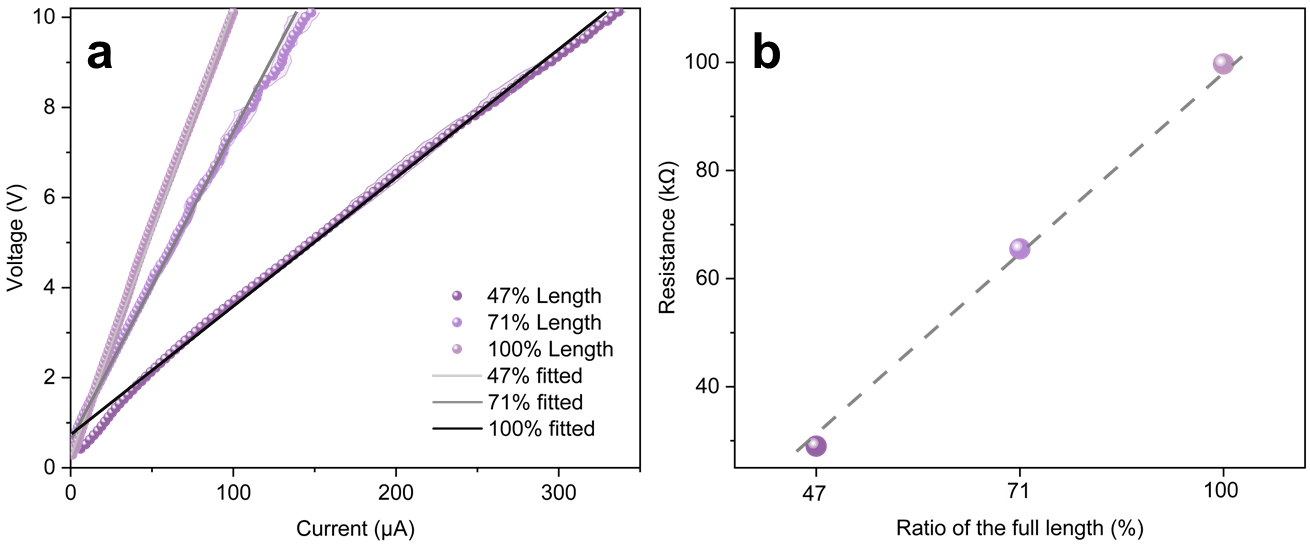
**

**Figure S14.** Conductivity of the liquid wires with different lengths. (a) Plot of the current of the circuit connected by the liquid wire with varied lengths as a function of DC voltages and the fitted curves. (b) The calculated resistance of the liquid wire obtained from (a).

**
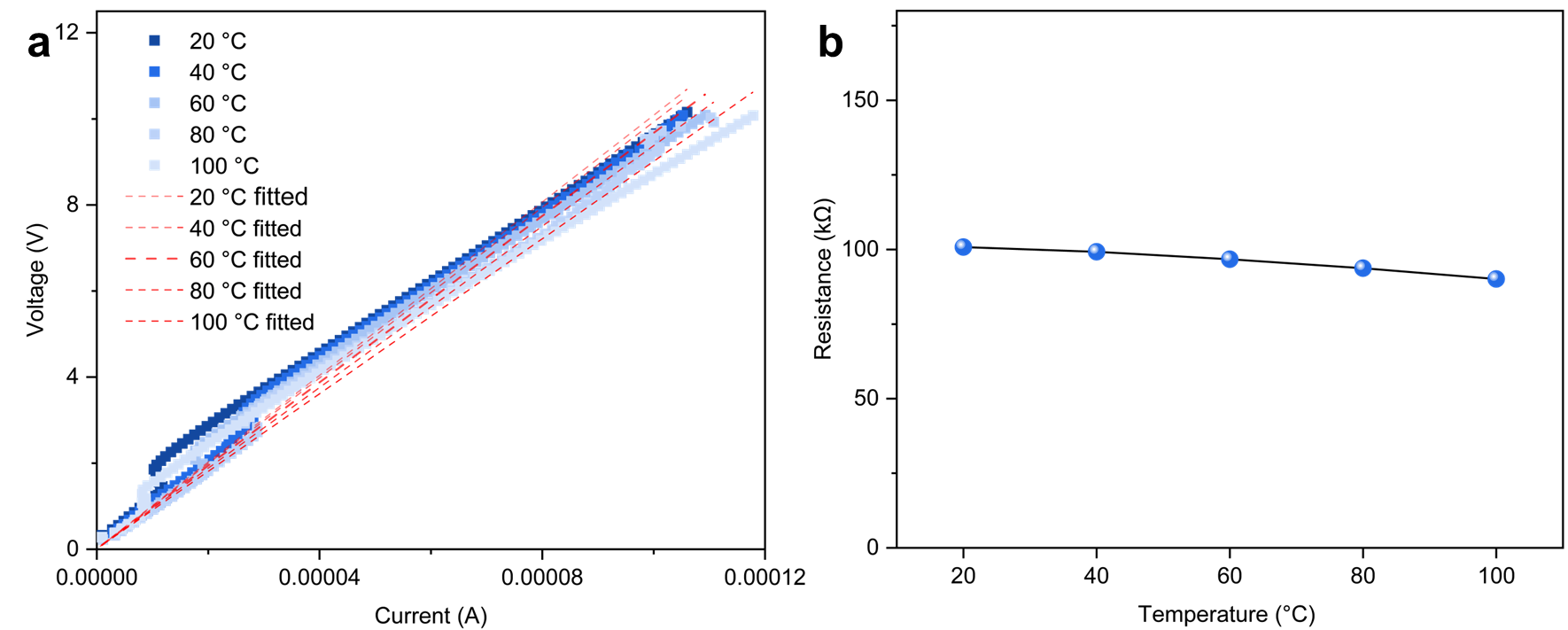
**

**Figure S15.** Thermal stability of the printed liquid tubular wire. (a) Plot of the current of the circuit connected by the liquid wire with temperatures as a function of DC voltages and the fitted curves. (b) Plot of the calculated resistance as a function of temperature.

**
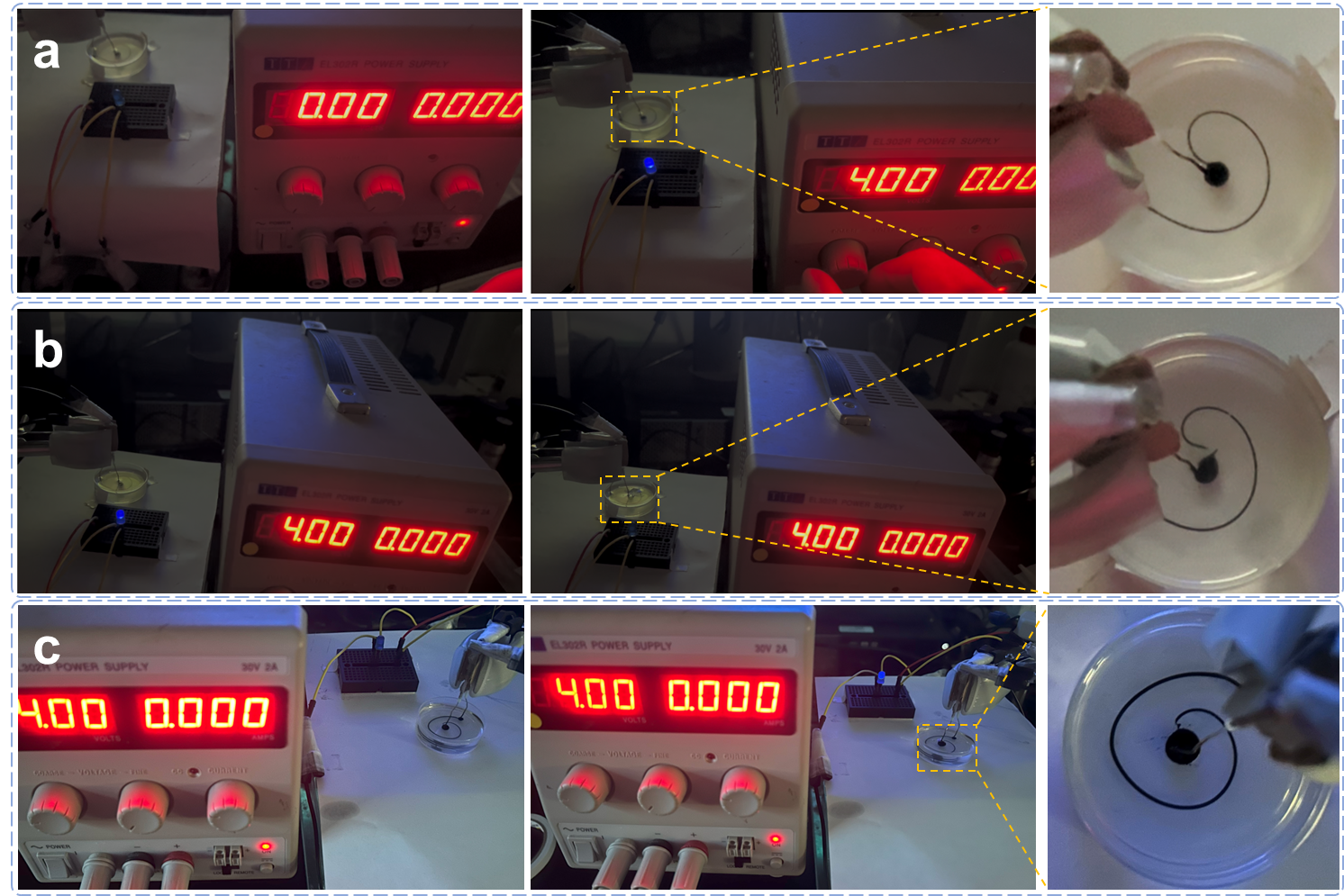
**

**Figure S16.** Demonstration for the liquid wire connecting the solid-state circuit. (a) Snapshots of the circuit connected by the printed liquid wire with the alight LED when increasing the voltage to 4 V. (b) Snapshots of the LED changing from bright to out after cutting off the wire under the voltage of 4 V. (c) Snapshots of the LED lighting up again after injecting the AuCl_4_^−^ solution into the place where the wire is broken under the voltage of 4 V.

**
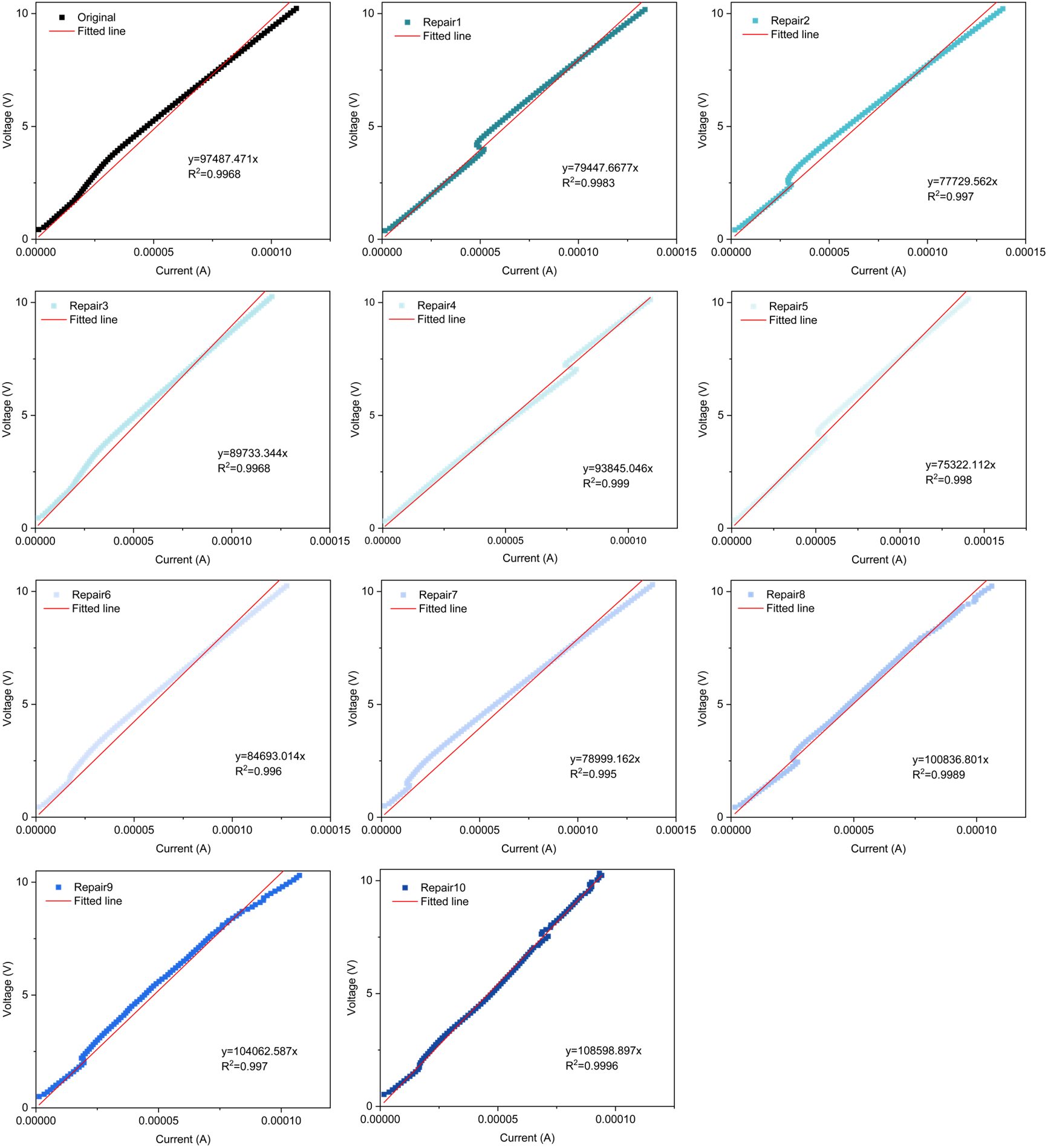
**

**Figure S17.** Profiles of the current changes as a function of DC voltage (0~10 V) in the circuit connected by the original printed wire and repaired 10-times wire.





**Figure S18.** Time-dependent currents of the DC circuit when the printed wire was cut under a constant DC voltage of 10 V.

**
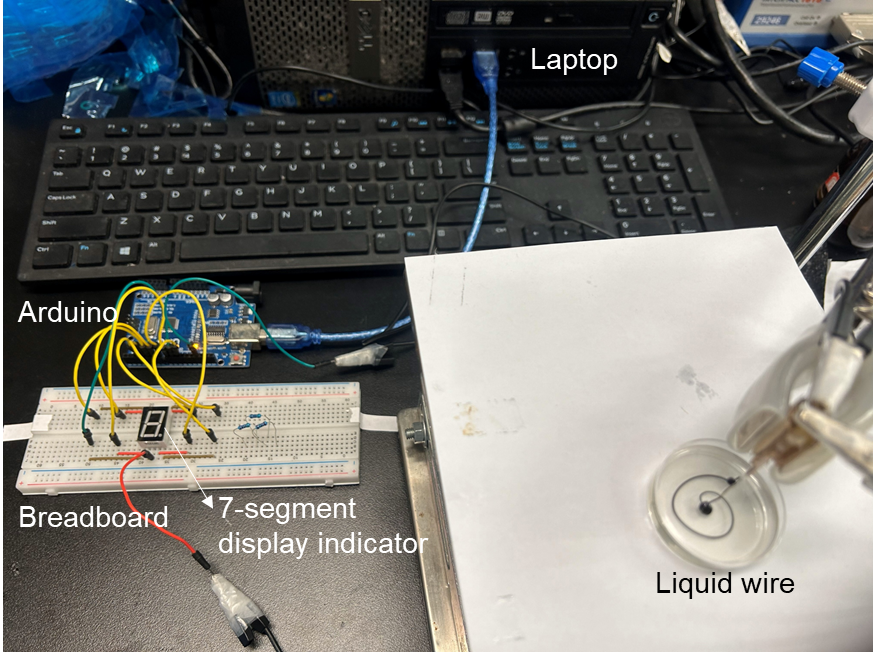
**

**Figure S19.** Actual snapshot of the experimental setup of the circuit connected by the liquid wire for information transmission.

**
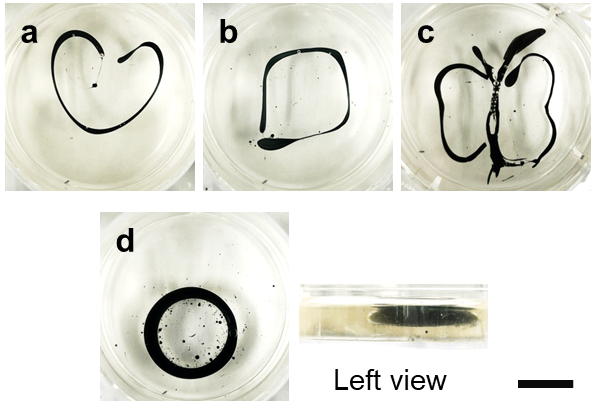
**

**Figure S20.** Liquid-in-liquid 3D printing of various strutures such as the heart shape (a), rhombus shape (b), butterfly (c), and the 3D circular ring (d). The ink of 5 mg mL^−1^ HAuCl_4_ and 10 mg mL^−1^ DSA solution was printed into silicone oil (60k mPa·S) containing 20 Py monomers at a 1200 mm min^−1^ moving speed and 500 μL min^−1^ flow rate.


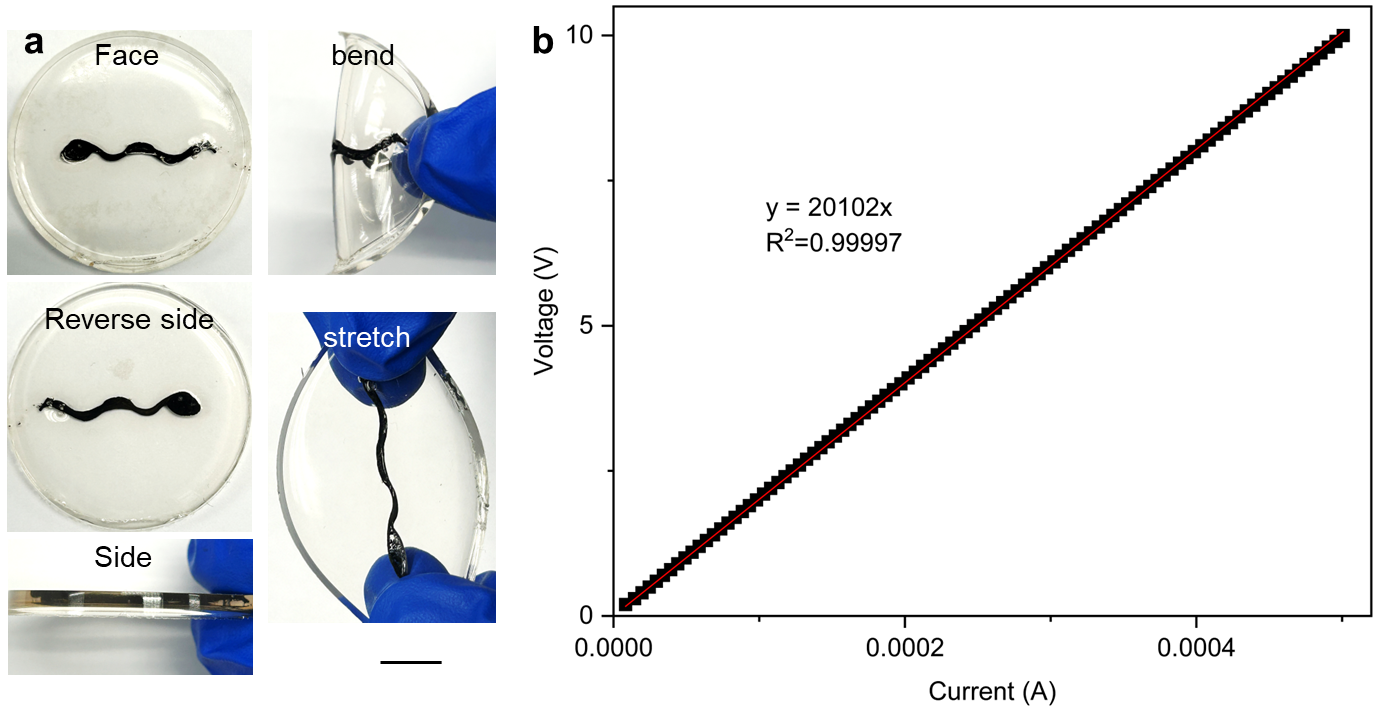


**Figure S21.** The intrinsic conductivity of the printed liquid tubular wire. (a) Snapshots of the liquid tubular wire in a cured silicone elastomer, showing the dried wire and adaptive mechanical property. (b) Profile of the current as a function of voltage of the circuit connected by the wire.
